# Local Anæsthetics

**Published:** 1894-11

**Authors:** L. E. Custer

**Affiliations:** Dayton, O.


### Local Anæsthetics.

BY L. E. CUSTER, D.D.S., DAYTON, O.

Read before the Northern Ohio Dental Society, June, 1894.

From the time that a deep sleep came over Adam, that one of his ribs might be painlessly removed for the well-being of Eve, agents have been employed in a great variety for producing insensibility to pain. Opium and hyoscyamus were used by the ancient Egyptians, and the heroes of Grecian myth produced wonders with hemp. Mandrake produced such profound slumber that a limb might be painlessly removed during its influence, and alcohol still possesses sufficient strength that a man may wake up in the morning with a bruised head and blackened eye and wonder how he came by them.

The whole subject of painless dentistry, including local anæsthetics, has been so exhaustively gone over by the Michigan State Society, just met, that little remains but what would be a repetition of its work, so instead of presenting an elaborate paper upon this subject, we will only touch upon a few of the more vital parts and close with what is known as Infiltration Anæsthesia.

Pain is a necessary safeguard of these bodies of ours and is so interwoven with the motor and special-sense functions of nerves that it can only be overcome with great danger to the vital processes, respiration and circulation. There are many operations in dentistry which involve a small area of sensitive tissue and which are quite painful enough to demand an anæsthetic of some kind, although hardly justifying the risk of a general anæsthetic. Such cases are met by the injection of an agent into the circulation at the point of operation, the topical application to the surface only which is absorbed by the underlying tissue and by refrigeration.

This not being a body of students it is unnecessary to go into methods and appliances as you are all familiar with them, but there is one point I would urge and that is the cleanliness of the needle and mouth. They must be aseptic. It is a recognized fact in surgery that there is more danger of septic poisoning from a puncture than from an open cut. In a hypodermic injection we not only have a puncture, but if we are not careful we have a puncture in a hot-bed of micro-organisms and their products. The needle must be aseptic and the mouth should have been washed with a germicide and disinfectant, none of which is more effective than bichloride of mercury $\frac{1}{1000}$ and peroxide of hydrogen equal parts.

Since the discovery of cocaine for local anæsthesia, there has been no single drug introduced of any marked value. Cocaine has become so popular that it is not only used alone but has become the base of nearly all formulæ for local anæsthesia. No single agent approaches it in potency and at the same time no agent seems to be quite so erratic in its action as cocaine, not as an anæsthetic but in its influence upon circulation and respiration. The needle may hardly be removed before the patient shows signs of syncope, palpitation or dyspnœa. Or on the other hand the operation may have been completed and in the midst of the most promising outcome, alarming symptoms of cocaine poisoning set in. These irregularities of cocaine are generally attributed to the idiosyncrasies of the patient, but it is my belief that quite as much is due to the irregular methods and quantities used in injection. At one time a large quantity may be used with apparently no disturbance whatever, and again a very small quantity may bring on alarming symptoms. To my mind this is easily explained. In one instance the contents may enter into the arterial circulation, when it will be quite as effective as an anæsthetic and produce no immediate signs of poisoning, while in another instance it may mostly enter the venous circulation, by which it is quickly carried from the desired point of action to the heart and lungs, thus transferring the action from the point of injection to the vital centers. If the alkaloid has entered the arterial system, the cocaine poisoning would be shown some time after the injection, while if it enters the venous system, these symptoms almost immediately follow as the result of injection into the arterial system, and is usually of a milder nature, because of greater dilution in the blood. It passes through the arteries and capillaries and is more largely diffused, so that it reaches the heart and lungs in less quantity. If the injection finds way more easily into the venous system it does not pass through the capillary circulation and quickly reaches the vital points in almost the pure state.

The combination of cocaine with other agents, I believe only adds to the danger without making it more effective. We do not know certainly what new agent may be formed by the addition of chloroform, chloral, carbolic acid or alcohol. One of the simplest and best formulæ is that given by Dr. N. S. Hoff:

| | | | |
|---|---|---|---|
| ℞ | Cocaine - - - - | gr. ss | |
| | Morphia Sulph. - - - | $\frac{1}{6}$ gr. | tab. 1 |
| | Atropine, - - - - | $\frac{1}{80}$ gr. | |
| | Carbolized water, 2 per cent. - | gtt xxv | |

Tropacocaine is a recent form for which much is promised. It seems to be an ideal anæsthetic, possessing all the good qualities of the muriate of cocaine and none of the bad.

While a topical application is not so effective as a hypodermic injection, it has an element of safety which recommends it. A heavier per cent. may be used than for injection, and when the membrane has been well dried with the warm air blast this method gives adequate results in superficial operations.

Before the administration of cocaine it is well to fortify the patient. Unlike with the use of a general anæsthetic a systemic antidote may be administered, without combating a local anæsthetic. A cup of strong coffee, or an injection of morphine may sustain the system through any irregularity.

Special attention is to be paid to respiration. If it ceases and the circulation is still going on, no matter how feebly, an agent may be injected into the circulation which will stimulate the respiratory centers; for this purpose an injection of $\frac{1}{10}$ grain strychnine is probably the best. For heart failure, medicaments may be inhaled, and the most effective for this purpose is nitrite of amyl, which paralyzes the inhibitory cardiac centers. An agent may also be injected for heart failure, but since it has to take effect through the circulation of the blood, it is evident that it can only avail in a weak pulse and is of no account whatever if the heart has entirely stopped. For this purpose an injection of ten to twenty drops of the tincture of digitalis is best.

Reduction of temperature is a local anæsthetic of no small importance. Where carefully used it is prompt, safe and effective. As a condition for the perfect performance of nerve function, there must be maintained a certain temperature, which is normal to the organism. If that temperature is lowered, neural activity is lessened and anæsthesia is produced in proportion to the departure from normality, so that it is quite possible to reach a point of complete anæsthesia or extreme numbness. It is not necessary to actually freeze the tissue, as is often done in deep operations. It is much better to operate slowly and repeat the application. A great variety of agents have been used for this purpose, but none are so well adapted for dental purposes as chloride of ethyl.

### INFILTRATION ANÆSTHESIA.

In 1891 the percentage of cocaine in solutions for injection was so low that it became a question as to whether pure water alone would not do as well. Sleich, of Berlin, was first to demonstrate that pure water in sufficient quantity, when injected, acted as a local anæsthetic. Many operators upon finding their cocaine solution exhausted or for other reasons, have since used water alone, with apparently as good results as when injecting cocaine solutions. The experiments of Sleich upon the injection of water for local anæsthesia, which he calls "*Infiltration Anæsthesia,*" when reported to the Berlin Medical Society, were accompanied by numerous cases and were enthusiastically received by Professor Virchow and the other members. A report says, "Over and over again this method of infiltration anæsthesia has been employed in hundreds of cases, simply because surgeons prefer infiltration anæsthesia to the anxiety of a chloroform narcosis, the dangers of which stand in no proportion to the importance of the operation."

The injection of pure water is painful and it is not until a minute after the injection that anæsthesia occurs. This is in a measure overcome by reducing the temperature by the ether or ethyl spray. The experiments showed that a number of agents might be used with the water to act as an anæsthetic, without producing irritation. The most effective was solutions of common salt. The various per cents. from two to six were tried, but the two per cent. gave the best results.

In explanation of this phenomena the author says, "The cause of anæsthesia by infiltration is not a simple one, several factors are at work: first, the pressure of the injected fluid and the removal of the blood from the infiltrated tissues. After a properly performed injection, they appear perfectly white. That is, instead of the tissue juices, the foreign mixture is incorporated in all the lymph vessels and areolar spaces, while the blood is gradually forced into the neighboring vessels. But besides this pressure and anæmia of the tissues, temperature plays a prominent part." A low temperature is more effective. Every œdema of the skin would be insensible did not the injected fluid, which causes the pathological œdema, contain the same amount of salt (six per cent.) as the serum of the nervous fluid. If one produces an artificial œdema with other fluids with or without a little common salt, the whole region of the artificial swelling will become insensible and will enable one to operate without causing pain. "This," he says "is briefly the principle of infiltration anæsthesia." When we study the methods ourselves, we find that in order that a nerve can communicate sensation, it must be constantly supplied with an amount of nutrient fluid which is normal to it, it must be in its natural investment and must be of a certain temperature. When the salt solution is injected it takes the place of the blood, so that the normal nutrition of the nerve ending is forced out. The solution differs largely from blood ; it is an abnormal surrounding. Again, according as the temperature is lowered the nervous reception and transmission of sensation is reduced.

In all the cases which present to us, he is the safe and wise operator who selects the local anæsthetic most suitable for the case.

If it is one of small depth he will use the least dangerous, a topical application or refrigeration, and only use injection when operating to some depth. The injection of a foreign substance into the circulation is a matter of no small importance and we should see to it that the agent is pure and the needle and membrane aseptic.

